# Beyond Membrane Fluidity: Lipid Unsaturation and Hofmeister Cations Govern Nanoplastic Dynamics at Membrane Interfaces

**DOI:** 10.1021/acs.analchem.6c02646

**Published:** 2026-08-26

**Authors:** Cristina Lopez-Galicia, Diyali Sil, Zhane Norris, Chayan Dutta

**Affiliations:** † Departments of Chemistry, 123425 Georgia State University, Atlanta, Georgia 30303, United States; ‡ The Center for Diagnostics and Therapeutics, Georgia State University, Atlanta, Georgia 30303, United States; § Molecular Basis of Disease, Georgia State University, Atlanta, Georgia 30303, United States

## Abstract

Interactions between plastic nanoparticles (PNPs) or nanoplastics and lipid membranes are governed by a coupled interplay of membrane composition and ionic environment, yet how lipid unsaturation and ion specificity regulate PNP diffusion remains poorly understood. Here, we investigate the effects of acyl-chain saturation and Hofmeister cations on the interactions of carboxylated polystyrene (PS) nanoparticles with phosphatidylcholine membranes composed of dipalmitoylphosphatidylcholine (DPPC), palmitoyloleoylphosphatidylcholine (POPC), and dioleoylphosphatidylcholine (DOPC) lipids. Langmuir isotherms show that lipid packing decreases with increasing unsaturation and that divalent cations, particularly Ca^2+^, induce pronounced membrane condensation across all lipid types, while monovalent cations produce weaker effects. To relate membrane structure to nanoparticle diffusion, we combine single-particle tracking (SPT) with fluorescence correlation spectroscopy super-resolution optical fluctuation imaging (fcsSOFI) as an analytical method to quantify nanoparticle confinement and diffusion on supported lipid bilayers. Salt addition modulates diffusion and confinement independently, in a manner that depends strongly on lipid identity. On POPC, confinement follows a divalent/monovalent distinction consistent with the Hofmeister series, while diffusion remains unchanged. On DPPC, diffusion is reduced by all salts without systematic confinement changes. On DOPC, both properties are largely insensitive to ion identity. These results show that nanoplastic dynamics at membrane interfaces cannot be predicted from membrane fluidity alone and are governed by the interplay between membrane phase state, mechanical compliance, and ion-specific headgroup interactions.

## Graphical abstract

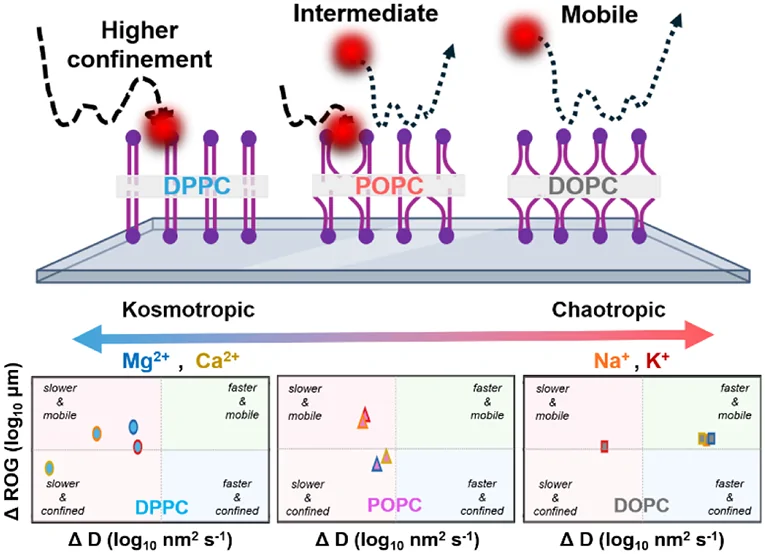

## Introduction

Plastic nanoparticles (PNPs) are increasingly detected in environmental and biological systems, raising concerns about their interactions with cellular membranes.^1–3^ At the nanoscale, particle–membrane interactions are governed not only by particle size and surface chemistry but also by the physicochemical properties of the membrane itself and the surrounding ionic environment.^4–7^ A fundamental challenge remains in understanding how membrane composition and ion-specific effects regulate nanoparticle adsorption, confinement, and mobility at membrane interfaces. Overcoming these lapses opens the potential to provide fundamental insights to the areas spanning nanomedicine, synthetic biology, and vaccine technologies, etc.^8–10^

Lipid acyl-chain unsaturation is a key determinant of membrane structure and dynamics.^8–10^ Increasing unsaturation introduces conformational disorder in lipid tails, leading to reduced packing efficiency, larger mean molecular area per lipid, and enhanced membrane fluidity. Fully saturated phospholipids form tightly packed ordered membranes, whereas unsaturated lipids adopt more expanded, fluid structures. These differences in lipid packing and phase state can influence how nanoplastics interact with membranes, affecting both their lateral mobility and degree of confinement at the membrane surface.^11,12^

In parallel, membranes are highly sensitive to their ionic environment. Specific cations interact with lipid headgroups in ways that can significantly alter membrane order. Divalent cations, particularly Ca^2+^, are known to bind phosphatidylcholine headgroups, reduce the area per lipid, and promote membrane condensation, while monovalent cations generally exert weaker and more indirect effects.^13,14^ These ion-specific interactions are often rationalized within the Hofmeister framework, which emphasizes the role of ion charge density, hydration, and specificity in modulating interfacial structure. The Hofmeister series ranks ions according to their hydration properties, ranging from strongly hydrated kosmotropic (Ca^2+^ and Mg^2+^) to weakly hydrated chaotropic (K^+^) ions. At membrane interfaces, cation hydration determines the nature and strength of ion-headgroup interactions.^15,16^ Despite extensive studies on ion-induced membrane ordering, how Hofmeister cations regulate nanoplastic dynamics on lipid membranes are not studied.

Lipid unsaturation and ionic effects do not act independently during nanoparticle–membrane interactions. Instead, nanoparticle behavior may emerge from a combination of membrane fluidity, lipid packing, ion-mediated headgroup interactions, and nanoparticle-surface adhesion. A highly fluid membrane may limit ion-induced condensation, while a more ordered membrane may amplify ion-specific effects, resulting in heterogeneous nanoparticle motion and confinement. The interplay between charged biomolecules and lipid membranes as mediated by cations has attracted considerable interest in membrane biophysics. Cation valency and identity have been shown to regulate the attachment and dynamics of charged macromolecules at lipid bilayers, with divalent cations facilitating bridging interactions at gel-phase membranes while monovalent cations modulate interfacial interactions through electrostatic screening.^17–19^ The phase state and acyl-chain chemistry of lipid membranes have emerged as key regulators of these cation-mediated interactions, with lipid tail unsaturation shown to modulate membrane surface charge and the propensity for interfacial binding beyond effects attributable to membrane fluidity alone. Disentangling these coupled effects requires analytical approaches that simultaneously probe membrane structure and nanoparticle dynamics with high spatial and temporal resolution. Our work contextualizes these insights to nanoplastic-membrane interactions, demonstrating that lipid acyl-chain unsaturation and Hofmeister cation identity jointly and independently regulate nanoparticle diffusion and confinement at phosphatidylcholine bilayers.

Here, we systematically investigate how lipid acyl-chain saturation and Hofmeister cations (K^+^, Na^+^, Ca^2+^, Mg^2+^) (Figure 1A–1B) jointly control the interactions of carboxylated polystyrene (PS) nanoparticles with model phosphatidylcholine membranes. Using Langmuir monolayers of dipalmitoylphosphatidylcholine (DPPC), palmitoyloleoylphosphatidylcholine (POPC), and dioleoylphosphatidylcholine (DOPC), we quantify ion-induced changes in lipid packing and correlate these structural effects with PNP dynamics on supported lipid bilayers using single-particle microscopy^5,20–22^ and fcsSOFI-based super-resolution analysis.^4,23,24^ This combined approach provides an analytical framework for understanding how membrane composition and ionic environment together govern nanoplastic-membrane interactions.

**Figure 1:**
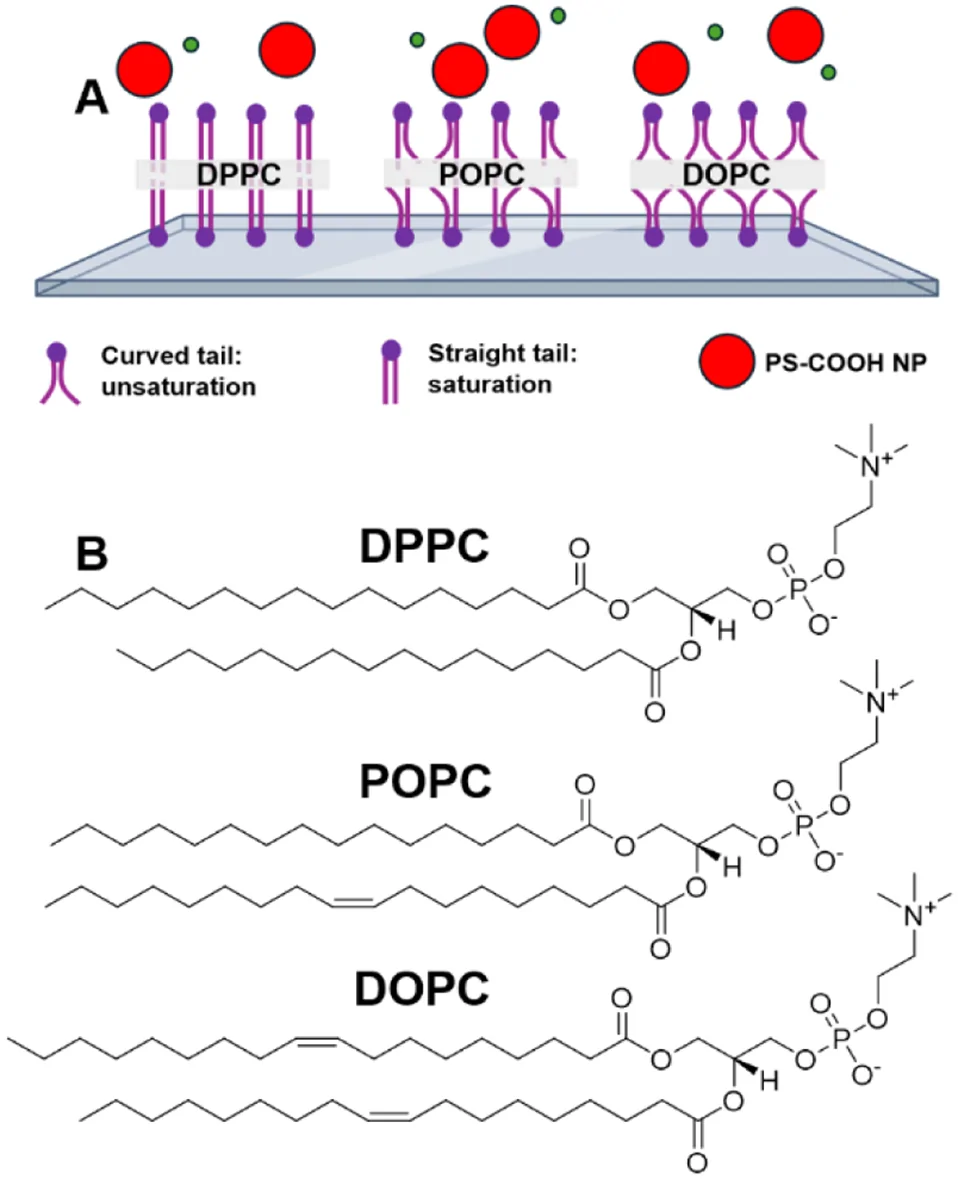
**A)** Schematic representation of supported lipid bilayers, composed of DPPC, POPC, and DOPC, each lipid having a different degree of unsaturation. Carboxylated PS nanoparticles interact with the lipids in the presence of different cations, M+ (green circles). **B)** Chemical structures of the lipids, DPPC, POPC, and DOPC. DPPC has no unsaturation, POPC has one unsaturated tail and one saturated tail, and DOPC has two unsaturated tail groups.

## Materials and Methods

### Vesicle Preparation

We prepared small unilamellar vesicles (SUV) using the vesicle fusion method.^25^ Lipids dissolved in chloroform solutions were deposited into 2 mL vials, dried under a gentle stream of air, and then placed into a desiccator to ensure the removal of any organic solvents. The drying time varied depending on the lipid type. DOPC and POPC thin films were placed in a desiccator for a minimum of 50 min, while DPPC thin films required overnight drying to ensure complete solvent removal. Afterward, a 5 mg/mL lipid solution was prepared in 20 mM HEPES buffer and then extruded using an Avanti-Mini Extruder (Avanti Polar Lipids, Inc.) through a polycarbonate membrane with a pore size of 100 nm. The extrusion process varies depending on the lipid. For both POPC and DOPC, we conducted the extrusion process at room temperature with no additional heating. DPPC SUVs were extruded and SLBs were formed at 55–60 °C, above the gel-to-liquid transition temperature (T_m_ ≈ 41 °C), to ensure complete vesicle fusion. All single-particle imaging experiments were performed at room temperature, at which DPPC bilayers are in the gel phase, POPC and DOPC bilayers are in the liquid-crystalline phase. This phase state is explicitly considered in the interpretation of nanoparticle dynamics throughout the manuscript. To ensure uniformity, all lipid suspensions were passed through the extruder 11–15 times to yield the consistently sized SUVs.

### Sample Preparation for Single Particle Measurements

Glass coverslips utilized for supported lipid bilayer (SLB) formation were cleaned by three sequential rounds of 15 min sonication in soapy water, methanol, and acetone. They proceeded to be immersed in a heated base piranha solution (5:5:25 H_2_O_2_, NaOH, and Millipore water) at 60–80 °C for 25 min. After cooling for 10–15 min, the coverslips were subjected to another 15 min sonication in Millipore water, which was then followed by air-drying. We treated the clean coverslips with oxygen plasma for 5 min. This plasma cleaning step hydrophilizes the surface, an essential property for supported lipid bilayer formation as the hydrophilicity of the glass induces vesicle fusion.

To prepare supported lipid bilayers, we attached a hybriwell well chamber to a plasma-cleaned coverslip. We then drop casted SUV solutions into the chamber in two 10 μL additions (20 μL total). The chamber was incubated for 90 min for DOPC and POPC, and 60 min for DPPC, to allow vesicle fusion and bilayer formation. Following incubation, we rinsed the chambers four times with 10 μL of 20 mM HEPES buffer (40 μL total). Once the SLB was ready, polystyrene nanoparticle solutions (∼7.2 × 10^10^ particles/mL) prepared with 200 μM salt was introduced into the chamber using a syringe pump at a flow rate of 200 nL/min.

### DLS and Zeta Potential Characterization of Polystyrene Nanoparticles

We used an Anton Paar Litesizer 500 Particle Analyzer to measure the size of the COOH-PS PNPs using a disposable cell. We prepared the samples in the same salt concentrations that we used for single-particle measurements. We used the same instrument to measure the surface charge of the COOH-PS PNPs using an Omega cuvette (Mat. No 225288).

DLS measurements (Figure S1A) show that particle sizes under all salt conditions (113–116 nm) are consistent with the manufacturer-reported value of ∼100 nm, with polydispersity index values below 0.1 in all cases, confirming that nanoparticles remain monodisperse and do not aggregate under the experimental conditions. Zeta potential measurements (Figure S1B) show that nanoparticles remain highly negatively charged under all conditions (−42 to −48 mV), with the exception of CaCl_2_, which produces a less negative zeta potential (≈−34 mV). This reduction likely reflects partial charge screening by Ca^2+^ due to its stronger headgroup coordination. Importantly, even under CaCl_2_ conditions, the zeta potential remains sufficiently negative to prevent aggregation.

### Langmuir Monolayer Preparation

Isotherm experiments were conducted using a Langmuir–Blodgett trough (KSV NIMA), with a surface area of 273 cm^2^. Prior to each experiment, the trough is cleaned with methanol, acetone, and Millipore water. For each trial, 80 μL of 1 mM lipid stock solution was spread onto the subphase. The subphase contained 150 mM salt solution in Millipore water. These conditions were consistent among all experiments. Before compression commenced, the system was equilibrated for 15 min. Barrier compression was carried out symmetrically at a speed of 8 mm/min.

### Compressibility Moduli Calculation

The compressibility modulus Cs^–1^ was calculated from the surface pressure–area isotherms as eq 1

1 $$C_{s}^{-1}=-A\frac{d\Pi }{dA}$$

where A is the mean molecular area at surface pressure Π. Cs^–1^ values provide a quantitative measure of monolayer mechanical stiffness^26^ and are used to identify lipid phase states where compressibility values of 10–50 mN/m correspond to the liquid-expanded (LE) phase, 50–100 mN/m to LE/LC coexistence, 100–250 mN/m to the liquid-condensed (LC) phase, and above 250 mN/m to the gel phase.^27,28^

### Data Analysis with SPT and fcsSOFI

Single particle data was analyzed through two main approaches, SPT and fcsSOFI.^23,24^ SPT links individual particle trajectories across consecutive image frames. Raw camera images were processed using a Gaussian filter to reduce background noise and enhance particle visibility, followed by radial symmetry fitting for subpixel accuracy.^29,30^

Fluorescence correlation spectroscopy super-resolution optical fluctuation imaging (fcsSOFI) was used to construct the diffusion maps of PNPs over lipid membranes.^4^ fcsSOFI combines two analytical techniques to resolve particle dynamics beyond the diffraction limit. The first, fluorescence correlation spectroscopy (FCS), measures temporal fluctuations to extract information about molecular diffusion. At equilibrium, fluorescent molecules will alter between dark and bright phases while also moving in and out of a small region, providing characteristic intensity fluctuations. By analyzing these signals, fcsSOFI quantifies diffusion behavior within localized regions. Data acquisition was performed using videos consisting of 7,000–10,000 frames and a frame interval of 0.01 s to capture nanoparticle diffusion dynamics. Temporal fluorescence fluctuations were modeled using the autocorrelation function described in eq 2.

The autocorrelation function compares signal variations at a given time with those at a later time lag (τ). This correlation reveals how rapidly molecules move within the observation area, elucidating the diffusion properties of PNPs.

2 $$G_{2}\left(r,\tau \right)=a\left(r\right)\left[\frac{1}{1+{\left(\tau /\tau_{D}\right)}^{\alpha }}\right]+b$$

Here, a­(r) represents the amplitude of the autocorrelation curve. *τ*_*D*_ represents the characteristic diffusion time. *α* is the anomalous diffusion exponent, describing the kind of diffusion, *b* represents a constant offset.

### Radius of Gyration Analysis

The radius of gyration (RoG) was calculated from the full two-dimensional trajectory of each tracked nanoparticle as eq 3

3 $$R_{g}=\sqrt{\frac{1}{N}\overset{N}{\underset{i=1}{\sum }}\left[{\left(x_{i}-\mathrm{x̀}\right)}^{2}+{\left(y_{i}-ỳ\right)}^{2}\right]}$$

where N is the number of positions in the trajectory, x_i_ and y_i_ are the particle coordinates at frame i, and *x̀* and *ỳ* are the trajectory mean positions. RoG values were log_10_-transformed after averaging and presented as cumulative distributions across all tracked trajectories.^24,31^ RoG describes the total spatial extent of each individual nanoparticle trajectory over its full observation period and therefore captures confinement behavior integrated over the entire trajectory.

### Single-Frame Displacement (SFD) Analysis

Single-frame displacements were calculated with the two-dimensional distance between consecutive particle positions separated by one frame interval (0.03 s) as eq 4

4 $$r=\sqrt{(x_{i+1}-x_{i}{)}^{2}+{\left(y_{i+1}-y_{i}\right)}^{2}}$$

Displacement values were plotted in log scale and pooled across all trajectories to construct probability distributions for each lipid and ionic condition.^21,32^ SFD distributions describe the population-level distribution of displacement magnitudes between consecutive frames, providing a snapshot of how far nanoparticles move at the single-step level. Because SFD pools displacements across all trajectories, it is sensitive to the relative proportions of mobile and confined populations but does not capture the full spatial extent of individual trajectories.

### van Hove Correlation Function

The self-part of the van Hove correlation function was computed as the probability distribution of particle displacements at a fixed time lag of 0.03 s, following the eq 5

5 $$G_{s}\left(\Delta x,\Delta t\right)=\frac{1}{N}\left\langle \overset{N}{\underset{i=1}{\sum }}\delta \left(x+x_{i}\left(t\right)-x_{i}\left(t+\Delta t\right)\right)\right\rangle$$

For each trajectory, displacements in x and y were calculated independently and pooled across all tracked particles to construct the distribution for each lipid system and ionic condition.^33,34^

## Results and Discussions

### Langmuir Isotherm

Lipid monolayer condensation depends on acyl-chain unsaturation and nature of cations, with Ca^2+^ exerting the strongest ordering effect.^35^ Langmuir surface pressure–area isotherms and compressibility moduli were measured for DPPC, POPC, and DOPC monolayers in the presence of 150 mM salt, consistent with physiologically relevant ionic concentrations,^36,37^ to assess how acyl-chain unsaturation and cation identity jointly govern lipid packing and membrane mechanical response (Figure 2).

**Figure 2:**
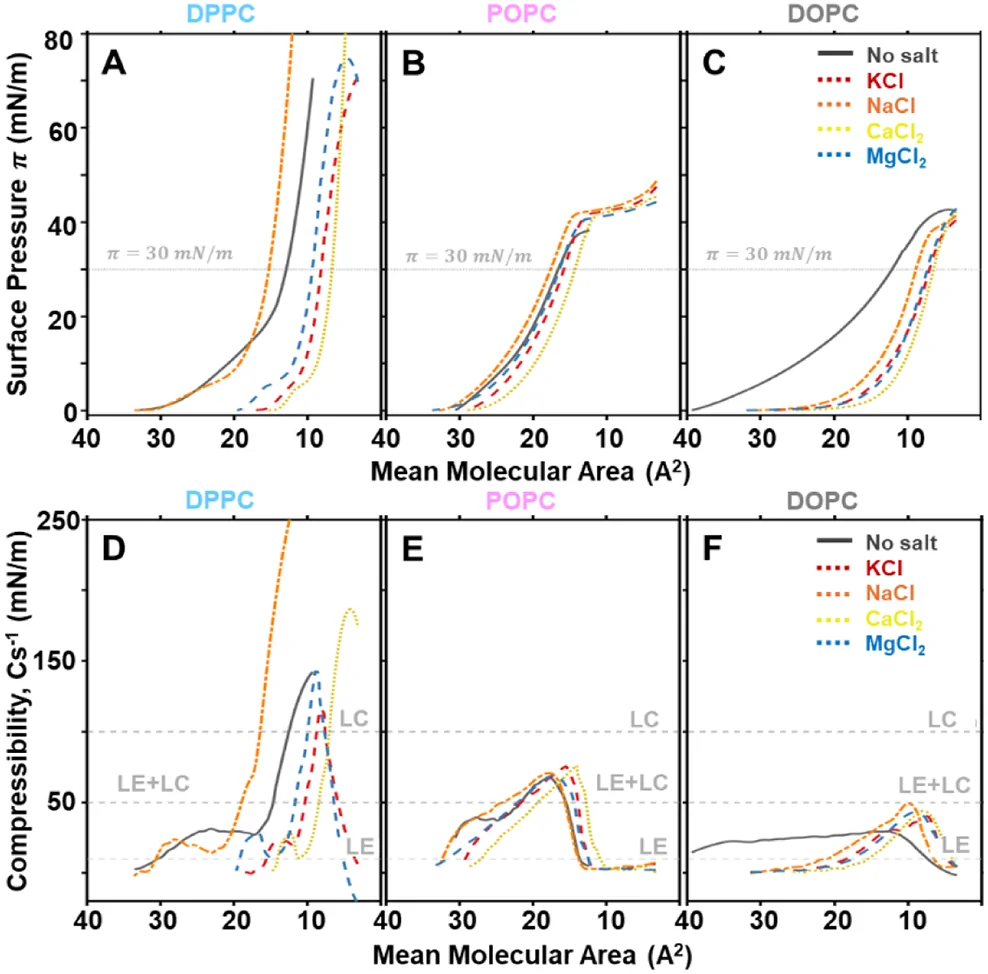
Langmuir monolayer characterization of DPPC, POPC, and DOPC under 150 mM salt conditions. Top row (A–C): Surface pressure–area isotherms for DPPC, POPC, and DOPC, respectively. The dotted horizontal line marks π = 30 mN/m, the physiologically relevant surface pressure. Bottom row (D–F): Compressibility modulus (Cs^–1^) as a function of surface pressure for the same systems. DPPC displays sharp, high-amplitude peaks indicative of cooperative phase transitions (LE → LE + LC → LC); POPC shows a broader, lower LC peak; DOPC exhibits no discernible peak, consistent with remaining in the LE phase throughout. Ion-induced changes in peak position and amplitude in DPPC reflect cation-specific modulation of monolayer mechanical stiffness, with effects diminishing in the more fluid POPC and DOPC systems.

In the isotherms (Figure 2A–2C), lipid packing in salt-free conditions reflects the degree of acyl-chain unsaturation. DPPC, with two saturated chains, forms the most condensed monolayer with a steep pressure rise at small molecular areas. POPC shows intermediate behavior, while DOPC exhibits the most expanded monolayer, initiating compression at the largest mean molecular areas with a gradual slope indicative of loosely packed lipids. These trends are consistent with previous reports.^38,39^ The compressibility modulus profiles (Figure 2D–2F) complement this picture where DPPC displays sharp, high-amplitude peaks corresponding to well-defined pressure-induced phase transitions (LE → LE + LC → LC), POPC shows a broader, lower LC peak, and DOPC exhibits no discernible peak, consistent with remaining in the LE phase throughout the accessible pressure range.

Salt addition produces ion- and lipid-specific shifts in both the isotherms and compressibility profiles, with both condensing and expanding effects observed depending on the specific cation and lipid system. To quantify these shifts, mean molecular area (MMA) values were extracted at π = 30 mN/m for each condition. Effects are most pronounced in DPPC and least pronounced in POPC. In DPPC (Figure 2A and 2D), ion-induced changes in MMA are the largest, ranging from 6.7 Å^2^ under CaCl_2_ to 15.3 Å^2^ under NaCl, compared to 12.9 Å^2^ in the salt-free condition. Ca^2+^ produces a strong condensing effect, consistent to its high charge density and reported coordination with lipid phosphate and carbonyl headgroups.^40–42^ Mg^2+^, K^+^, and Na^+^ exhibit progressively weaker condensing effects, following the order Ca^2+^ > Mg^2+^ ≈ K^+^ > Na^+^, consistent with the Hofmeister series and the influence of cation hydration. Notably, NaCl produces an expansion relative to the no-salt condition, highlighting that ion-induced shifts are not uniformly leftward. In POPC (Figure 2B & 2E), Mg^2+^ and K^+^ closely overlap with the salt-free condition (MMA ∼ 16.6 Å^2^), indicating minimal perturbation to lipid packing, while Ca^2+^ (MMA ∼ 14.3 Å^2^) and Na^+^ (MMA ∼ 17.7 Å^2^) produce slight shifts showing narrower spread than DPPC.^43^ In DOPC (Figure 2C and 2F), all salts reduce MMA substantially relative to the no-salt condition (from 12.2 Å^2^ to 6.5–8.9 Å^2^), though no systematic ion-specific ordering is observed across the salt conditions, consistent with the loosely packed, highly expanded nature of DOPC monolayer.^13^ Ca^2+^ consistently produces the smallest MMA across all three lipid systems, confirming it as the strongest condensing cation.

The surface pressure of π = 30 mN/m is widely used as the reference condition for comparing Langmuir monolayer properties to bilayer behavior, as previous work suggests that the lateral pressure experienced by lipids in a bilayer corresponds to this range.^26,44,45^ We acknowledge that supported lipid bilayers formed by vesicle fusion can be subject to substrate-induced effects and incomplete fusion.^26^ To assess bilayer quality, fluorescence imaging was performed under no salt and 200 μM salt conditions, confirming the formation of continuous, defect-free bilayers in each case (Figures S2 and S3). Substrate effects on lipid mobility are well documented, particularly for gel-phase bilayers such as DPPC, and may attenuate absolute diffusion values compared to free-standing bilayers. The compressibility modulus Cs^–1^ is therefore used here as a relative mechanical parameter to compare lipid systems and ionic conditions, rather than as a quantitative predictor of bilayer properties.

### Single-Frame Displacement and van Hove Distribution

PS nanoparticle diffusion on different supported lipid bilayers was imaged using TIRF microscopy and analyzed by SPT as described in the Methods Section. For supported lipid bilayer experiments, nanoparticle solutions were prepared at 200 μM salt. We have shown before that higher salt concentrations cause carboxy-PS nanoparticles to adsorb irreversibly to the bilayer surface.^5^ While the two concentrations used for Langmuir and single particle experiments are not equivalent, previous studies on lipid membranes have shown that ion-specific effects on lipid packing are approximately linear at low concentrations, with the qualitative ordering between divalent and monovalent cations preserved across this concentration range used in this study.^15^ The Langmuir monolayer data therefore serve to establish the qualitative structural response of lipid headgroups to specific cations, which is then related to nanoparticle dynamics on supported bilayers. On all lipid surfaces and under all conditions examined, three types of nanoparticle motion were observed, Brownian diffusion, confined diffusion, and immobile particles. Representative trajectories illustrating these behaviors are presented in Figure S4.

To assess nanoparticle mobility on supported lipid bilayers, SFD distributions were measured for PS nanoparticles on DPPC, POPC, and DOPC in salt-free conditions (Figure 3A). The distributions reveal a clear dependence on lipid composition. DPPC exhibits a bimodal distribution, with a distinct confined population at smaller displacements and a mobile population at larger displacements, indicating the coexistence of two dynamically distinct nanoparticle populations on the condensed-phase bilayer. For POPC, the amplitude of the confined population is smaller and shifted toward the longer population compared to DPPC. DOPC displays unimodal distribution with only longer displacements, consistent with the higher fluidity of the DOPC bilayer. SFD distributions measured under different salt conditions revealed ion-induced changes in displacement magnitudes. However, no systematic ion-specific ordering was observed in either the confined or mobile nanoparticle populations (Figure S5). SFD samples all nanoparticles displacements including mobile, confined, and immobile populations. Ion-specific effects that might selectively modulate a subpopulation of confined particles are therefore can be averaged making systematic trends difficult to resolve. van Hove correlation functions computed at a time lag of 0.03 s (Figure 3B) where DOPC exhibits the broadest distribution, reflecting greater nanoparticle displacement over the given time lag. POPC and DPPC show narrower distributions, with DPPC displaying the most restricted displacement profile. Together, these results show that nanoparticle dynamics on lipid bilayers depend on membrane composition and phase state, with the condensed-phase DPPC bilayer showing a distinct confined nanoparticle population. We also note the existence of a narrower center peak in the van Hove distribution, showing that some level of particle confinement also exists in the POPC and DOPC lipid membranes, but the degree of confinement is lower compared to the DPPC membrane. A qualitative schematic of nanoparticle motion on lipids in salt free conditions are shown in Figure S6.

**Figure 3:**
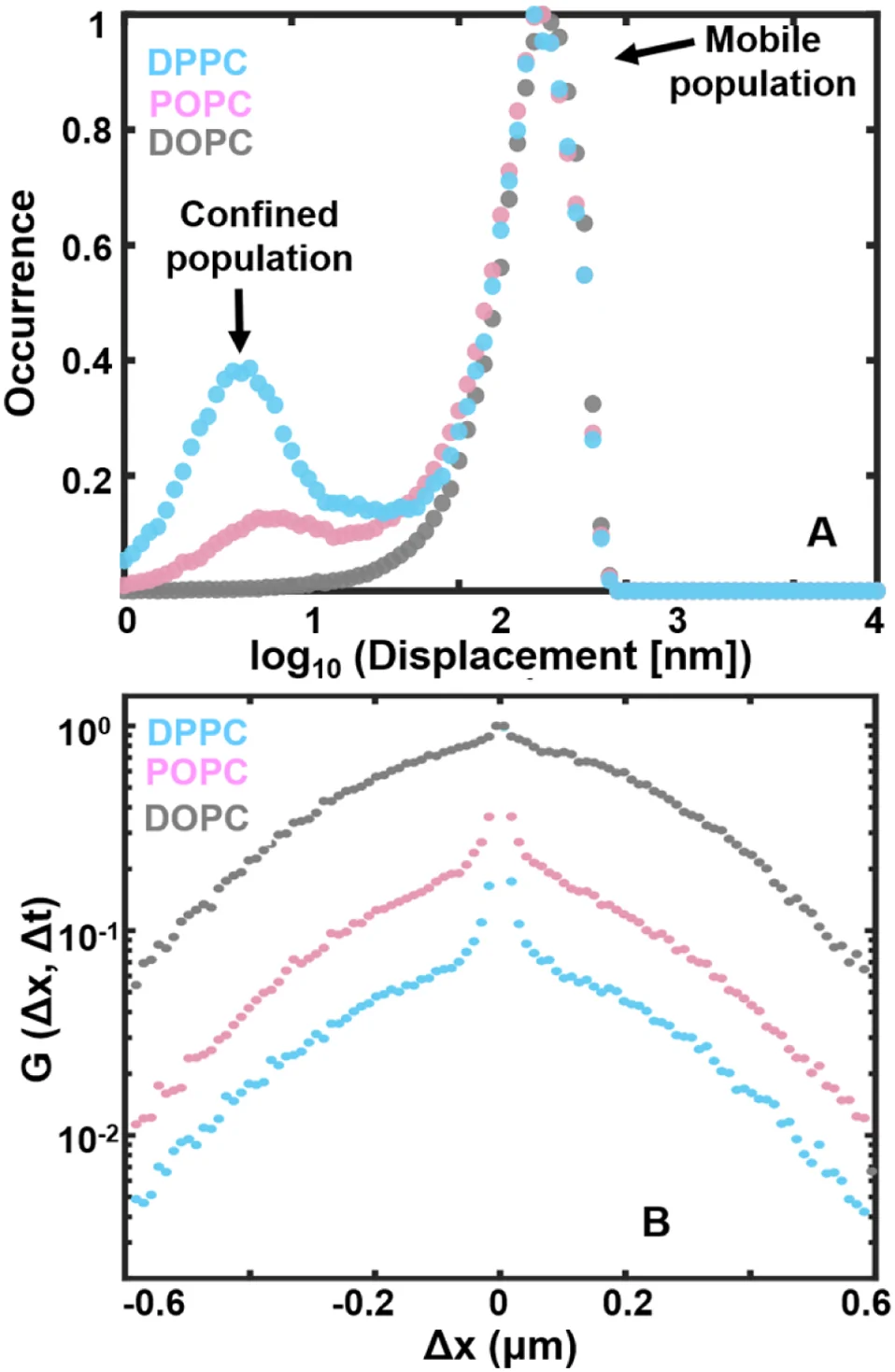
Single-frame displacement and van Hove analysis of PS nanoparticles on salt-free supported lipid bilayers. A) Log displacement distributions for DPPC, POPC, and DOPC. DPPC displays a bimodal distribution revealing coexisting confined and mobile nanoparticle populations. B) van Hove correlation functions at a time lag of 0.03 s. DOPC shows the broadest displacement distribution, DPPC the narrowest, and POPC intermediate behavior.

### Radius of Gyration Analysis

The spatial extent of nanoparticle trajectories was quantified using the RoG, which measures how far a particle explores from its center of mass. Cumulative distributions of log_10_(RoG/μm) for PS nanoparticles on salt-free supported lipid bilayers (Figure 4A) reveal that nanoparticle confinement follows the order POPC > DPPC > DOPC, with mean log_10_(RoG/μm) values of 0.64, 0.81, and 0.88, respectively. This order does not follow membrane fluidity, which ranks DPPC < POPC < DOPC. Nanoparticles on LC phase DPPC (*T*_m_ ≈ 41 °C) explore a larger area than on fluid-phase POPC (*T*_m_ ≈ −2 °C), suggesting that membrane fluidity alone does not determine nanoparticle confinement.^46–48^ Nanoparticle–membrane adhesion and headgroup interactions likely contribute independently to the observed confinement behavior.^38,49^

**Figure 4:**
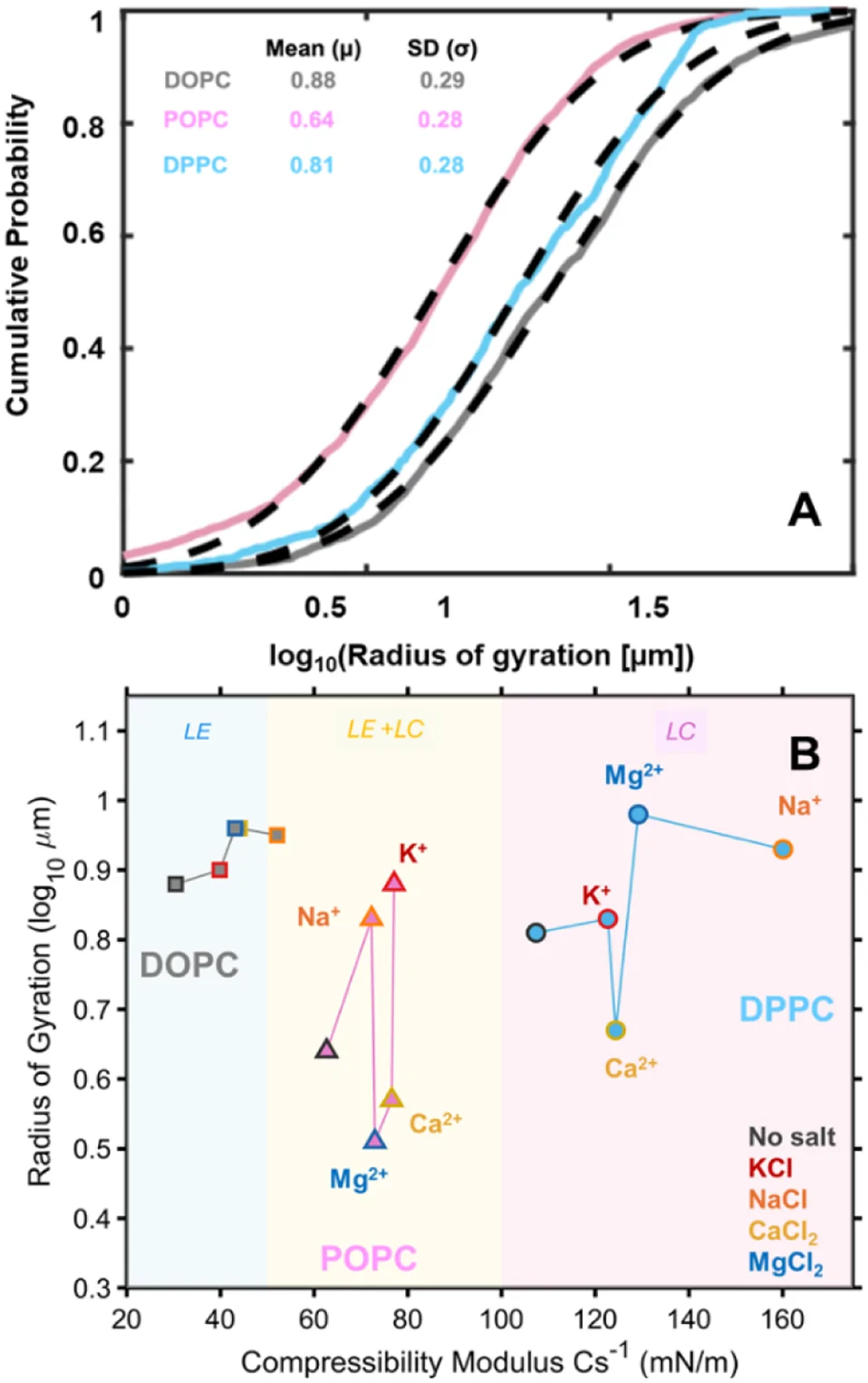
Radius of gyration analysis of PS nanoparticles on supported lipid bilayers. A) Cumulative distributions of log_10_(RoG/μm) for DPPC, POPC, and DOPC in salt-free conditions. Dashed lines are fits. Mean and standard deviation values are indicated. B) Mean log_10_(RoG/μm) plotted against compressibility modulus Cs^–1^ at π = 30 mN/m for all lipid and salt conditions.

Salt-dependent RoG distributions (Figure S7) reveal distinct ion-specific responses across the three lipid systems. On DOPC, all ionic conditions produce near-identical distributions relative to the no-salt condition, with mean RoG values ranging narrowly from 0.88 to 0.96, indicating that nanoparticle confinement on this bilayer is insensitive to cation identity. On POPC, divalent cations strongly enhance confinement, Mg^2+^ and Ca^2+^ yield the smallest mean RoG values (0.51 and 0.57 respectively), while monovalent cations reduce confinement relative to no salt, with K^+^ and Na^+^ yielding mean RoG values of 0.88 and 0.83. This divalent/monovalent distinction is consistent with the Hofmeister series. On DPPC, no clear ion-specific trend is observed across most conditions, though Ca^2+^ produces a notable reduction in mean RoG to 0.67 compared to 0.81 in salt-free conditions, consistent with its strong condensing effect on DPPC monolayers (Figure 2A).^14^ Mg^2+^ yields the largest RoG among the salts (0.98), consistent with previous reports suggesting that Mg2+ retains a strongly hydrated shell near lipid bilayers, limiting direct headgroup coordination.

To examine whether membrane mechanical properties underlie these ion-specific responses, mean RoG values are plotted against the compressibility modulus Cs^–1^ measured at π = 30 mN/m for each lipid and salt condition (Figure 4B).^50^ Phase state boundaries (LE, LE+LC, LC) are defined based on literature ranges for Cs^–1^ as described in the Methods Section. The three lipid systems occupy distinct regions of this plot corresponding to their phase state, DOPC in the LE phase at Cs^–1^ < 50 mN/m, POPC in the mixed LE + LC phase at Cs^–1^ between 50 and 100 mN/m, and DPPC in the LC phase at Cs^–1^ > 100 mN/m. DOPC data points cluster tightly regardless of ionic condition, confirming ion-insensitive confinement on this highly compliant membrane. POPC shows the largest spread in RoG across ionic conditions despite a narrow range of Cs^–1^ values, indicating that ion identity strongly modulates nanoparticle confinement on membranes of intermediate mechanical stiffness without proportionally altering monolayer compressibility. DPPC data points span a broader range of Cs^–1^ but show less systematic variation in RoG, with the exception of Ca^2+^ which produces the smallest RoG at intermediate Cs^–1^. The carboxylated PS nanoparticles carry negative surface charge in all the conditions used here, as confirmed by zeta potential measurements (Figure S1B). Electrostatic interactions between these negatively charged nanoparticles and the zwitterionic PC headgroups are therefore expected to be modulated by the ionic environment. Divalent cations such as Ca^2+^ and Mg^2+^ can act as electrostatic bridges between negatively charged nanoparticle surfaces and lipid headgroups through cation-mediated bridging.^17^ This mechanism is consistent with our observation that Ca^2+^ consistently enhances nanoparticle confinement across all lipid systems.

### Diffusion Coefficient Distributions from fcsSOFI Analysis

Nanoparticle diffusion coefficients were measured across supported lipid bilayers using fcsSOFI, which generates spatially resolved diffusion maps. Representative diffusion maps (Figure S8) of mobile, confined, and immobile nanoparticle populations are shown in the Supporting Information, and such type of diffusion has been observed across all lipid systems and ionic conditions, consistent with the SPT analysis described above. The fcsSOFI diffusion coefficient is derived from pixel-wise temporal fluorescence fluctuations within localized membrane regions and reflects local nanoparticle–membrane interaction dynamics. It does not necessarily correlate with the single step translational mobility captured by SFD or the spatial confinement captured by RoG.

Cumulative distributions of diffusion coefficients in salt-free conditions (Figure 5A) reveal that nanoparticle diffusion follows the order DPPC > POPC > DOPC, with mean log_10_(D/nm^2^ s^–1^) values of 5.55 (σ = 0.31), 5.47 (σ = 0.35), and 5.21 (σ = 0.43), respectively. This ordering is counterintuitive from a membrane fluidity standpoint. DOPC, the most fluid bilayer, yields the slowest nanoparticle diffusion, while DPPC yields the fastest. These results are consistent with the RoG analysis and suggest that nanoparticle–membrane adhesion contributes independently to translational mobility. One possible explanation is that the rigid DPPC surface limits interfacial contact with PS nanoparticles, reducing adhesion and allowing faster diffusion, while the fluid DOPC surface may promote stronger nanoparticle–membrane interaction, slowing lateral motion.^11^

**Figure 5:**
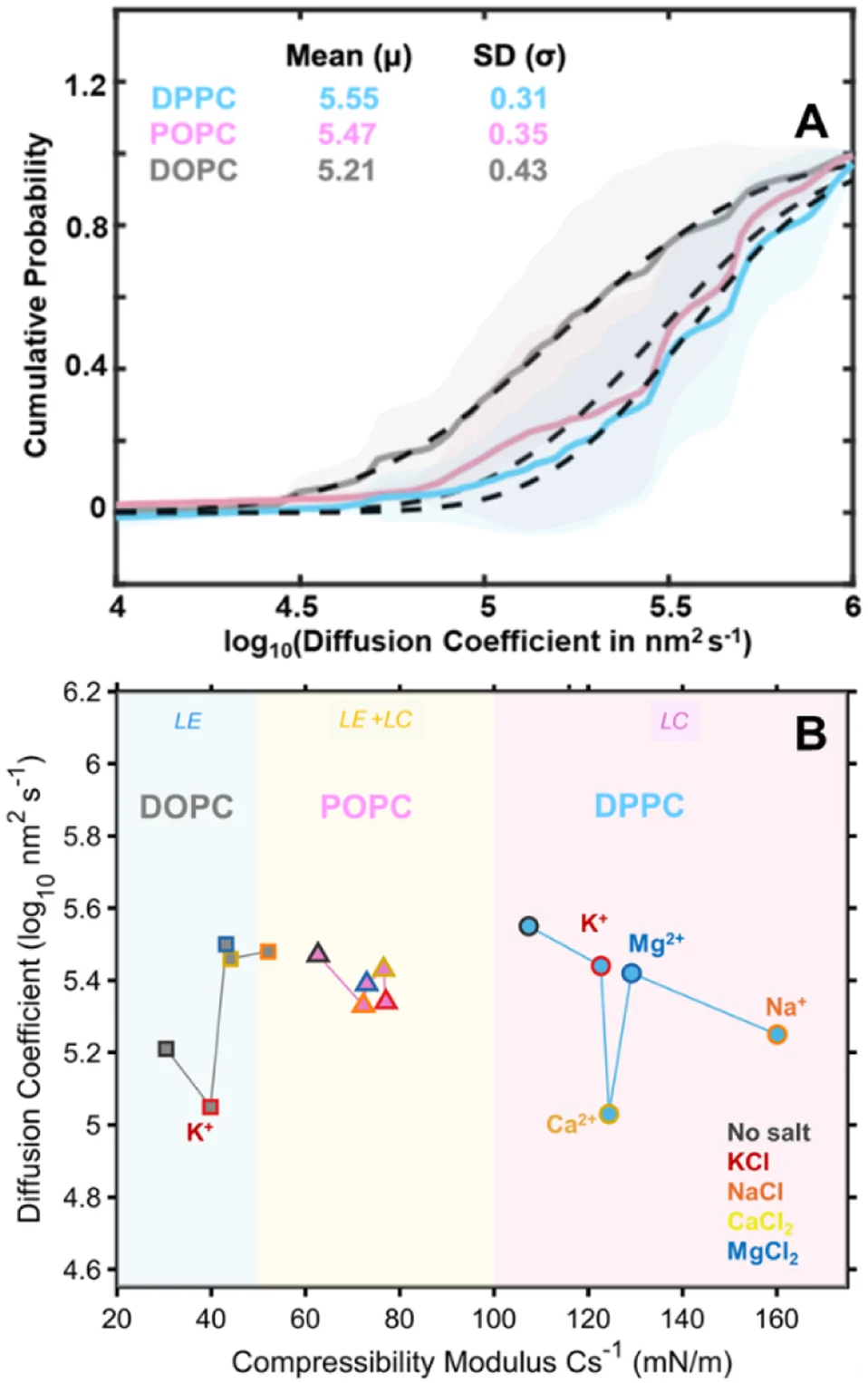
Diffusion coefficient analysis of PS nanoparticles from fcsSOFI. A) Cumulative distributions of log_10_(D/nm^2^ s^–1^) for DPPC, POPC, and DOPC in salt-free conditions. Dashed lines are fits. Mean and standard deviation values are indicated. Shaded regions represent standard deviation from 5 different data sets. B) Mean log_10_(D/nm^2^ s^–1^) plotted against compressibility modulus Cs^–1^ at π = 30 mN/m for all lipid and salt conditions.

To examine whether membrane mechanical properties underlie these differences, mean diffusion coefficients are plotted against compressibility modulus Cs^–1^ at π = 30 mN/m for all lipid and salt conditions (Figure 5B). The three lipid systems occupy distinct phase regions, DOPC in the LE phase (Cs^–1^ < 50 mN/m), POPC in the LE + LC phase (Cs^–1^ between 50 and 100 mN/m), and DPPC in the LC phase (Cs^–1^ > 100 mN/m). DPPC spans the broadest range of Cs^–1^ values with corresponding variation in diffusion coefficients, whereas POPC and DOPC shows minimum variation. Salt-dependent diffusion coefficient distributions are presented in Figure S9. In DPPC (Figure S9A), all salts reduce diffusion relative to no salt, with Ca^2+^ producing the largest reduction (mean 5.03) and Mg^2+^ the smallest (mean 5.42). K^+^ produces a bimodal distribution with populations at 4.59 and 5.44, indicating coexistence of two dynamically distinct nanoparticle populations. In POPC (Figure S9B), salts similarly reduce diffusion relative to no salt (mean 5.47), with Ca^2+^ producing the strongest effect and a bimodal distribution with populations at 4.20 and 5.43. In DOPC (Figure S9C), most salts increase diffusion relative to no salt (mean 5.21), with Na^+^, Ca^2+^, and Mg^2+^ yielding similar mean values near 5.46–5.50, while K^+^ reduces diffusion to 5.05. The absence of a consistent ion-specific ordering in DOPC is consistent with the RoG analysis, which showed ion-insensitive nanoparticle behavior on the highly compliant DOPC membrane.

Nanoparticle diffusion and spatial confinement respond independently to ionic conditions in a manner that depends strongly on lipid identity. To illustrate this, changes in mean diffusion coefficient (ΔDiff) and radius of gyration (ΔROG) relative to salt-free conditions are plotted for each cation and lipid system (Figure 6). The four quadrants of each panel distinguish qualitatively different behavioral responses, slower and more confined, slower and more mobile, faster and more confined, and faster and more mobile. On DPPC (Figure 6A), all salts reduce diffusion relative to no salt while changes in confinement are small and show no systematic ordering across cations. Ca^2+^ produces a slight increase in confinement while Mg^2+^, Na^+^, and K^+^ produce slightly positive ΔROG. On POPC (Figure 6B), monovalent cations increase RoG and divalent cations decrease it, while diffusion coefficients remain close to the no-salt value across all ionic conditions. Here confinement responding strongly to ion identity while diffusion remains largely unchanged, demonstrates that the two properties are independently modulated by ionic conditions for POPC membrane. On DOPC (Figure 6C), salts increase diffusion (except for K^+^) with minimal effect on confinement, with data points clustered near ΔROG ≈ 0 regardless of cation identity, consistent with the ion-insensitive behavior observed for DOPC lipid in the RoG analysis.

**Figure 6:**
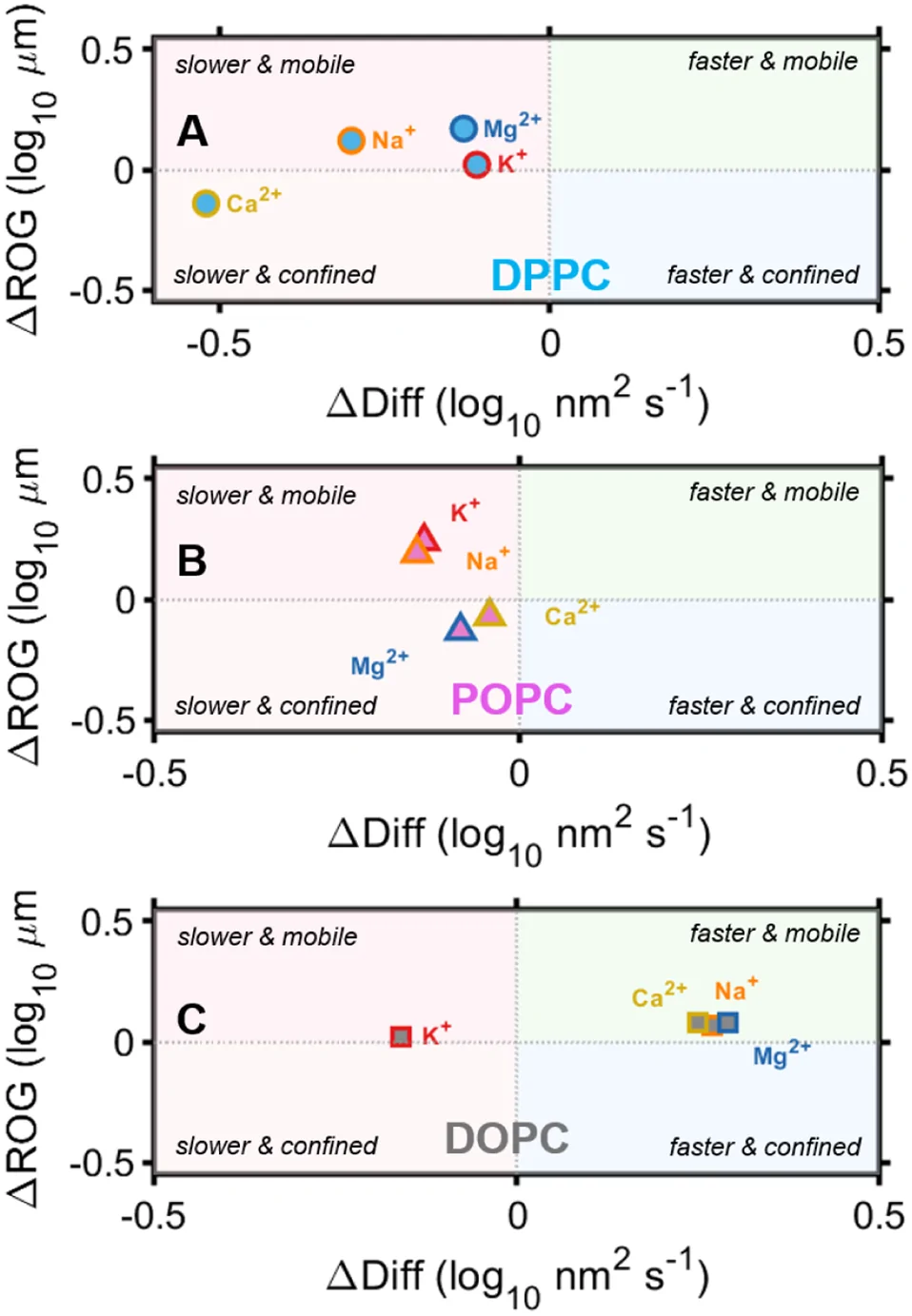
Decoupling of ion-induced changes in nanoparticle diffusion and confinement. Changes in mean diffusion coefficient (ΔDiff, log_10_ nm^2^ s^–1^) and radius of gyration (ΔROG, log_10_ μm) relative to salt-free conditions for PS nanoparticles on DPPC (A), POPC (B), and DOPC (C).

Across all three lipid systems, diffusion and confinement do not change synchronously in response to ionic conditions. On POPC, confinement follows a divalent/monovalent distinction consistent with the Hofmeister series, while diffusion is insensitive to ion identity. On DPPC, diffusion is reduced by all salts without systematic confinement changes. On DOPC, diffusion increases modestly while confinement remains unchanged.

To isolate the contribution of acyl-chain unsaturation from membrane phase effects, we compare POPC and DOPC directly, as both lipids are in the liquid-crystalline phase at room temperature. POPC contains one saturated and one monounsaturated acyl chain, while DOPC contains two monounsaturated chains. Despite sharing the same phase state, POPC exhibits stronger nanoparticle confinement (mean RoG = 0.64) than DOPC (mean RoG = 0.88) in salt-free conditions and shows pronounced ion-specific response in confinement while DOPC is largely ion-insensitive across all conditions. These differences cannot be attributed to phase state alone and suggest that increasing acyl-chain unsaturation modulates nanoparticle–membrane interactions. The more compliant DOPC bilayer limits the sensitivity of nanoparticle dynamics to ionic conditions, while POPC (less compliant than DOPC) allows ion-specific effects to manifest, consistent with recent findings linking acyl-chain unsaturation to membrane surface charge and interfacial interactions.^51^ The diffusion coefficients reported here are not measures of lipid lateral diffusion but the diffusion of the nanoparticles. Substrate-induced reduction in lipid lateral diffusion in SLBs therefore does not directly affect the reported observables. The differences observed between DOPC and POPC bilayers are consistent with their intrinsic composition-dependent packing properties. For fluid-phase bilayers, the thin interfacial hydration layer (∼10–20 Å) between the bilayer and the glass support is expected to preserve the liquid-crystalline phase state at room temperature.^10^ These observations indicate that the response of nanoparticle dynamics to ionic conditions is governed by the interplay between membrane phase state, mechanical compliance, and ion-specific headgroup interactions, rather than by any single parameter.

We also note that the degree to which PS nanoparticles interact with the membrane surface may differ across lipid systems. Tightly packed, gel-phase lipids such as DPPC present a rigid interface characterized by all-trans acyl chain conformation and tight lateral packing, which may limit the contact area between the nanoparticle surface and the lipid headgroups.^52,53^ In contrast, fluid membranes such as DOPC, with loosely packed and more disordered acyl chains, may allow greater conformational adaptation at the interface, increasing the effective contact between the nanoparticle and the membrane. We note that resolving nanoparticle contact depth or perpendicular motion at the membrane interface is beyond the capabilities of our current TIRF-based imaging approach used in this study, and direct measurement of interfacial coupling remains an important direction for future work.

## Conclusions

Here we demonstrate that nanoplastic-membrane interactions are regulated by a coupled interplay between lipid acyl-chain saturation, membrane mechanical compliance, and ion-specific headgroup interactions, rather than by membrane fluidity alone. Langmuir monolayer measurements show that lipid packing decreases with increasing acyl-chain unsaturation. Divalent cations, particularly Ca^2+^, condense all three lipid monolayers, while monovalent cations have weaker and more lipid-dependent effects. Membrane mechanical compliance, quantified by the compressibility modulus Cs^–1^, might be a key parameter governing ion-sensitivity where highly compliant membranes such as DOPC buffer ion-induced changes in nanoparticle dynamics and confinement, while membranes of intermediate compliance such as POPC are most responsive to cation identity.

Nanoparticle dynamics on supported lipid bilayers, quantified by SPT and fcsSOFI analysis, reveal a consistent departure from fluidity-based expectations. In salt-free conditions, nanoparticle diffusion follows the order DPPC > POPC > DOPC, and nanoparticle confinement follows the order POPC > DPPC > DOPC, neither of which corresponds to the membrane fluidity ranking. These results indicate that additional factors, including nanoparticle–membrane adhesion, may contribute to the observed dynamics. The coexistence of mobile, confined, and immobile nanoparticle populations across all lipid systems and conditions further underscores the heterogeneous nature of nanoparticle–membrane interactions.

Salt addition modulates nanoparticle dynamics in a manner that depends strongly on lipid identity. Ca^2+^ consistently reduces nanoparticle diffusion across all lipid systems, consistent with its strong direct coordination with phosphate and carbonyl headgroups leading to membrane condensation.^13^ Mg^2+^ and monovalent cations produce weaker and more lipid-dependent responses, reflecting differences in cation hydration and headgroup binding affinity.^42^ On POPC, nanoparticle confinement follows a divalent/monovalent distinction consistent with the Hofmeister series, while diffusion remains largely insensitive to ion identity. On DPPC, diffusion is reduced by all salts without systematic confinement changes. On DOPC, diffusion increases while confinement remains unchanged across all ionic conditions.

We also demonstrate that diffusion and confinement do not respond synchronously to ionic conditions instead they can be independently modulated depending on lipid identity (such as in POPC). This decoupling, combined with the departure from fluidity-based expectations, shows that nanoplastic behavior at membrane interfaces cannot be predicted from any single physicochemical parameter. Instead, it emerges from the interplay between membrane phase state, mechanical compliance, and ion-specific headgroup interactions. These findings suggest that membrane mechanical properties and ionic environment should be considered alongside lipid composition when assessing nanoplastic behavior in complex biological or environmental systems.

## Supplementary Material



## Notes

DLS and zeta potential characterization of polystyrene nanoparticles; representative membrane images; schematic illustration of nanoparticle motion in salt-free conditions; Representative PS trajectories; single-frame displacement distributions for all salts; Radius of gyration (ROG) for all salts; representative fcsSOFI diffusion maps; distribution of diffusion coefficient (PDF)
