# Supplementary material for: Beyond Membrane Fluidity: Lipid Unsaturation and Hofmeister Cations Govern Nanoplastic Dynamics at Membrane Interfaces
Source: Anal Chem. 2026 Aug 26;98(35):25711–20. doi: 10.1021/acs.analchem.6c02646 (PMC13564036; doi:10.1021/acs.analchem.6c02646)
Supplement: Supplementary file 1 [file ac6c02646_si_001.pdf]

Supporting Information

Beyond Membrane Fluidity: Lipid Unsaturation and Hofmeister Cations Govern Nanoplastic Dynamics at Membrane Interfaces

Cristina Lopez-Galicia[1], Diyali Sil[1,3], Zhane Norris[1], Chayan Dutta[1,2]*

[1]Departments of Chemistry, Georgia State University, Atlanta, GA 30303, USA.

[2]The Center for Diagnostics and Therapeutics, Georgia State University, Atlanta, GA 30303, USA.

[3]Molecular Basis of Disease, Georgia State University, Atlanta, GA 30303, USA.

*** Corresponding Author**:

*To whom correspondence should be addressed: Chayan Dutta, Department of Chemistry, Georgia State University, P.O. Box 3965, Atlanta, GA 30302-3965, U.S.A.

Phone: (404) 413-5530

EMAIL: cdutta@gsu.edu

Keywords: Nanoplastic transport, Single particle tracking, fcsSOFI, Hofmeister cations

Table of contents:

1. DLS and zeta potential characterization of polystyrene nanoparticles S3
2. Representative membrane images S4
3. Representative PS trajectories S6
4. Single frame displacement distributions for all salts S7
5. Schematic illustration of nanoparticle motion in salt free condition S8
6. Radius of gyration (RoG) for all salts S9
7. Representative fcsSOFI diffusion maps S10
8. Distribution of diffusion coefficient S11

1. DLS and zeta potential characterization of polystyrene nanoparticles

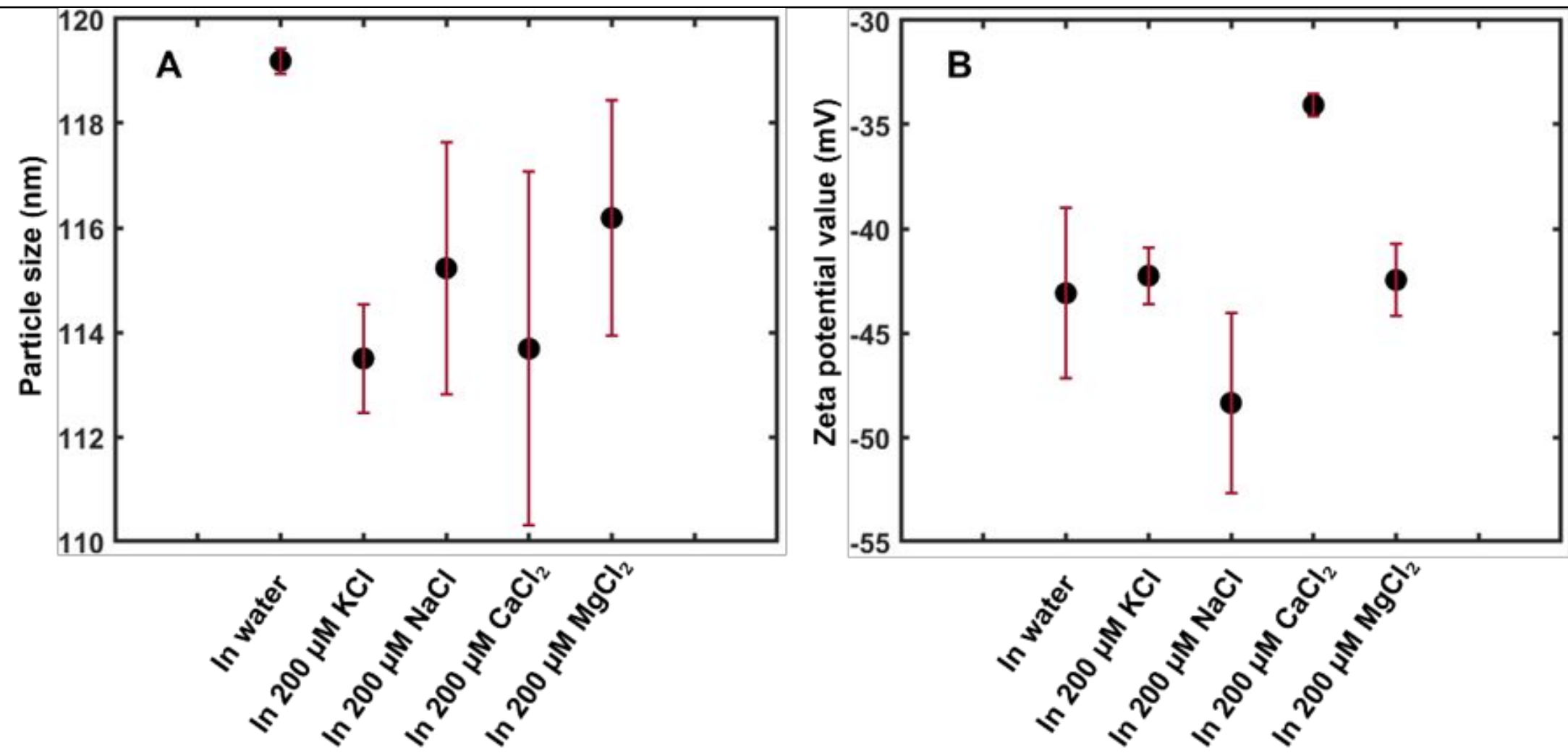

Figure S1: A) DLS measurements and **B)** zeta potential measurements of PS particles prepared in different types of salts. The error bar indicates the standard deviation from 3 individual measurements.

2. Representative membrane images

Fluorescence imaging of SLBs was performed for all three lipid systems under no salt and, 200 µM NaCl and $CaCl_2$ salt conditions. The images confirm the formation of continuous bilayers without visible defects (Figure S2). These data demonstrate that observed differences in nanoparticle dynamics across lipid types and ionic conditions reflect genuine lipid- and ion-dependent effects rather than artifacts of bilayer preparation. We acknowledge that some sample to sample variation might exist in the SLB quality and that might induce limited impact on the single particle results, however, we also performed controlled single particle experiment on the bare plasma cleaned glass substrate in our previous work to show that the PS PNP interaction on the glass surface is negligible compared to the SLB surfaces.[1]

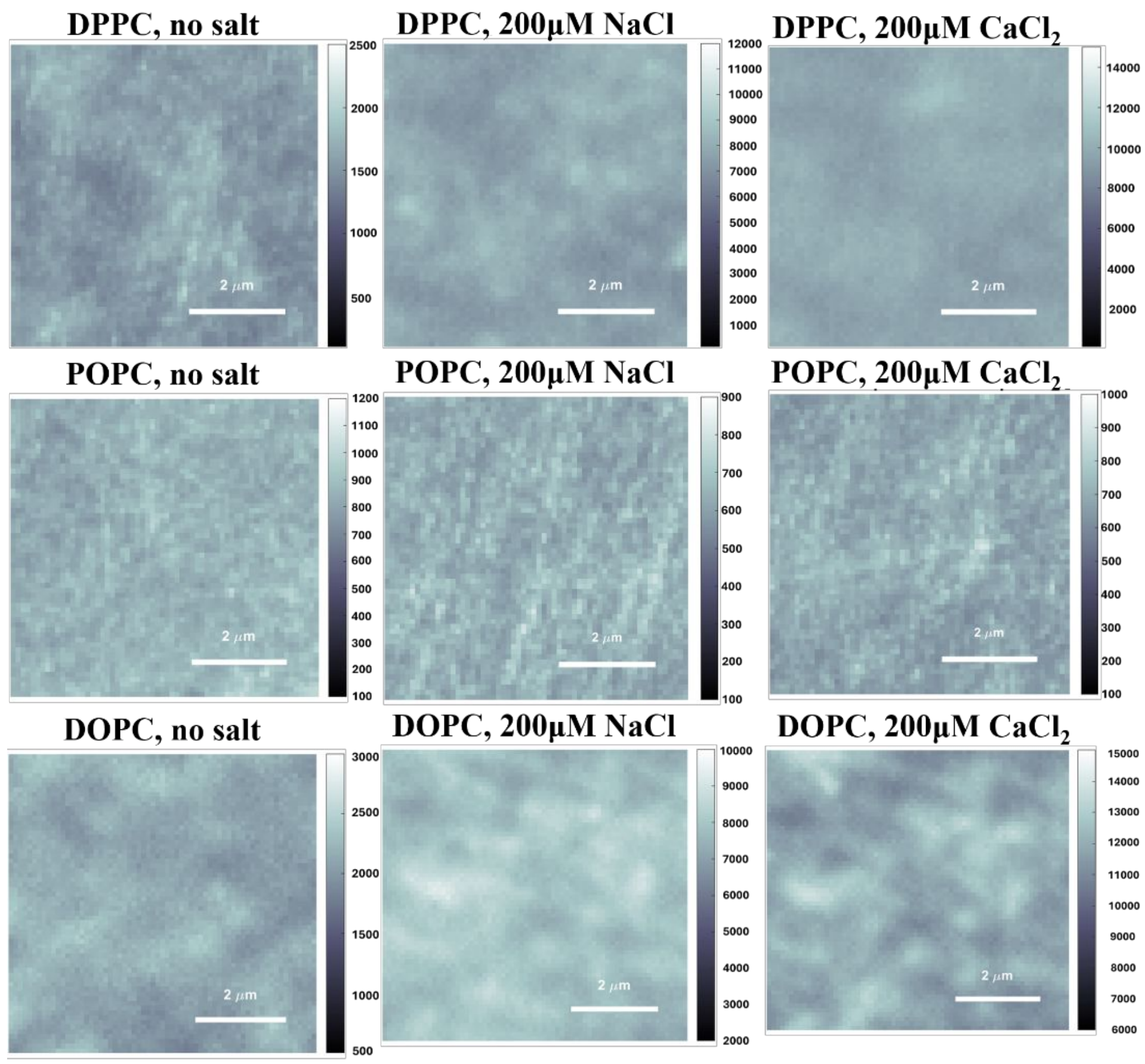

Figure S2: Uniform coverage of NR on SLB without salt and in presence of 200 µM salts, showing a homogeneous lipid bilayer on plasma cleaned coverslip.

Averaged horizontal line intensity profiles were extracted from each image and normalized to the mean image intensity. The absence of sharp intensity dips and the narrow standard deviation across all line profiles confirm continuous, defect-free bilayer formation under all conditions examined (Figure S3).

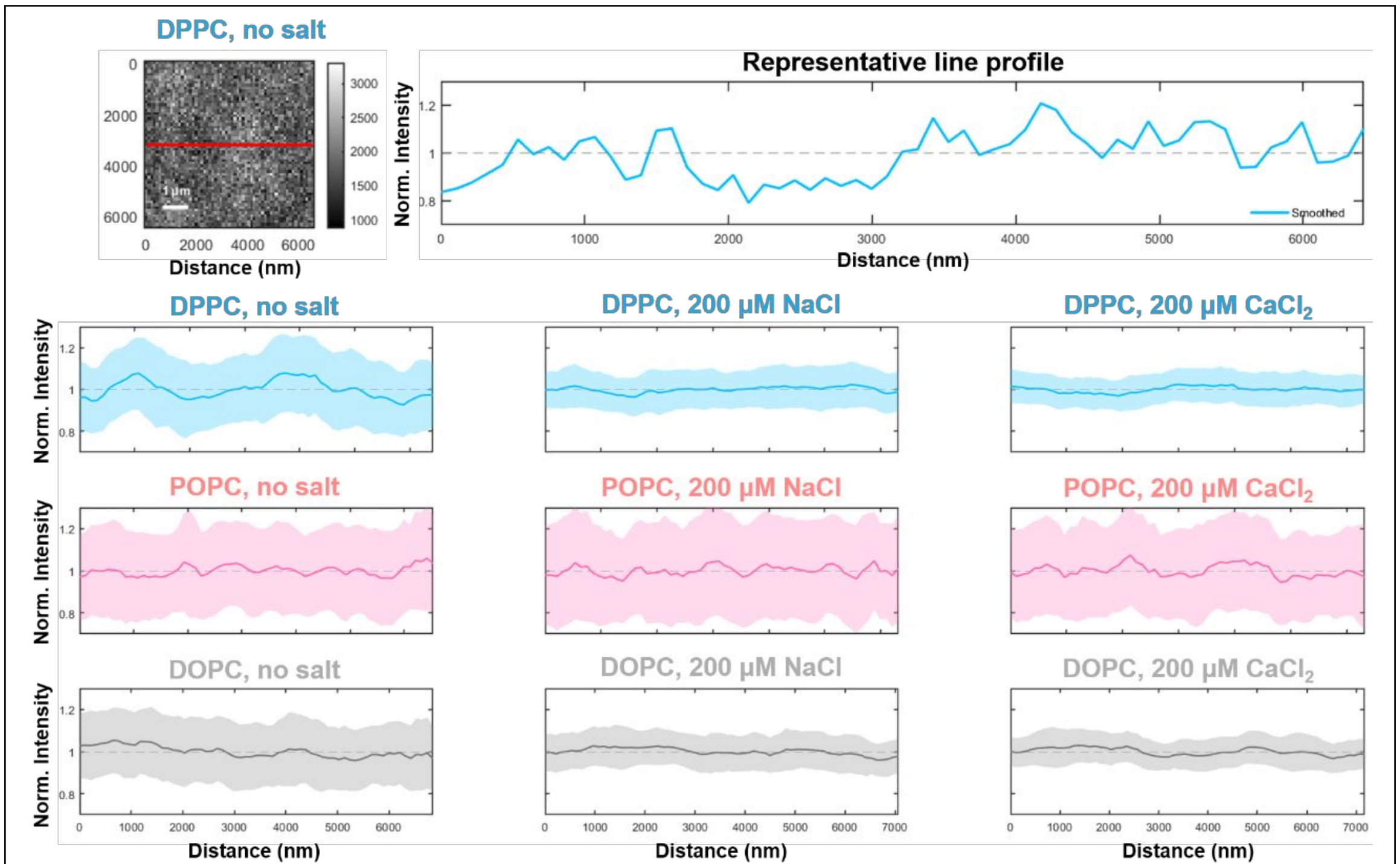

Figure S3: Line profile analysis of supported lipid bilayers. Top left: representative fluorescence image of a DPPC bilayer in the absence of salt. The red line indicates a representative horizontal line section. Top right: normalized intensity profile of the representative line (gray) showing uniform fluorescence and its variation along the normalized maximum intensity across the horizontal line. Lower panels: averaged normalized fluorescence intensity profiles for all lipid systems (DPPC, POPC, DOPC) and ionic conditions (no salt, 200 µM NaCl, 200 µM $CaCl_2$). Solid lines represent the mean normalized intensity averaged across all horizontal line profiles within each image. Shaded regions represent ± one standard deviation. The absence of sharp intensity dips and narrow standard deviation bands across all conditions confirm continuous, spatially uniform bilayer formation. Pixel size: 107 nm. Scale bar in representative image: 1 µm.

3. Representative PS trajectories

Here are the representative trajectories observed for PS nanoparticles on the lipid surfaces. For each type of lipid system, we analyzed atleast 5 different ROIs and selected more than 20000 trajectories.

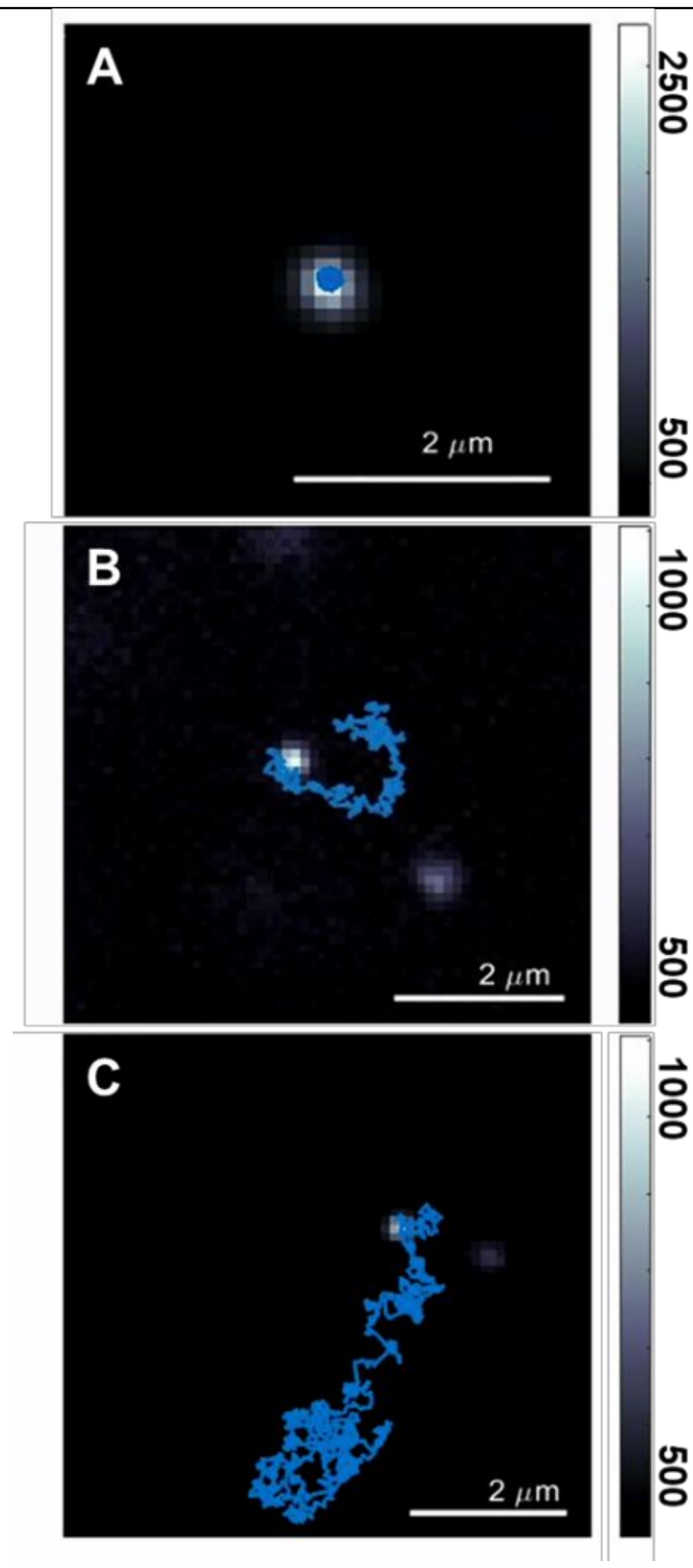

Figure S4: Representative types of trajectories illustrating three types of motion of PS nanoparticles observed over all three lipids. Confined (A), intermediate (B), and Brownian (C) type diffusion trajectories.

4. Single frame displacement distribution of all salts

Single-frame displacement of the PS nanoparticles was calculated over the supported lipid bilayer (SLB) under salt conditions. For each type of lipid, in the presence of each type of salt condition, we analyzed more than 2000 trajectories from atleast 5 ROIs after first frame filtering.[1] Figure S5 shows the SFD distributions for all the cations at each lipid surface. From these observed trends it is difficult to correlate if there was any specific trends for long and short populations as a function of lipid unsaturation. Nevertheless, these observations highlight the intricate and ion-dependent nature of nanoparticle-membrane interactions, where both bilayer phase state and specific cation-headgroup interactions contribute to the observed displacement heterogeneity.

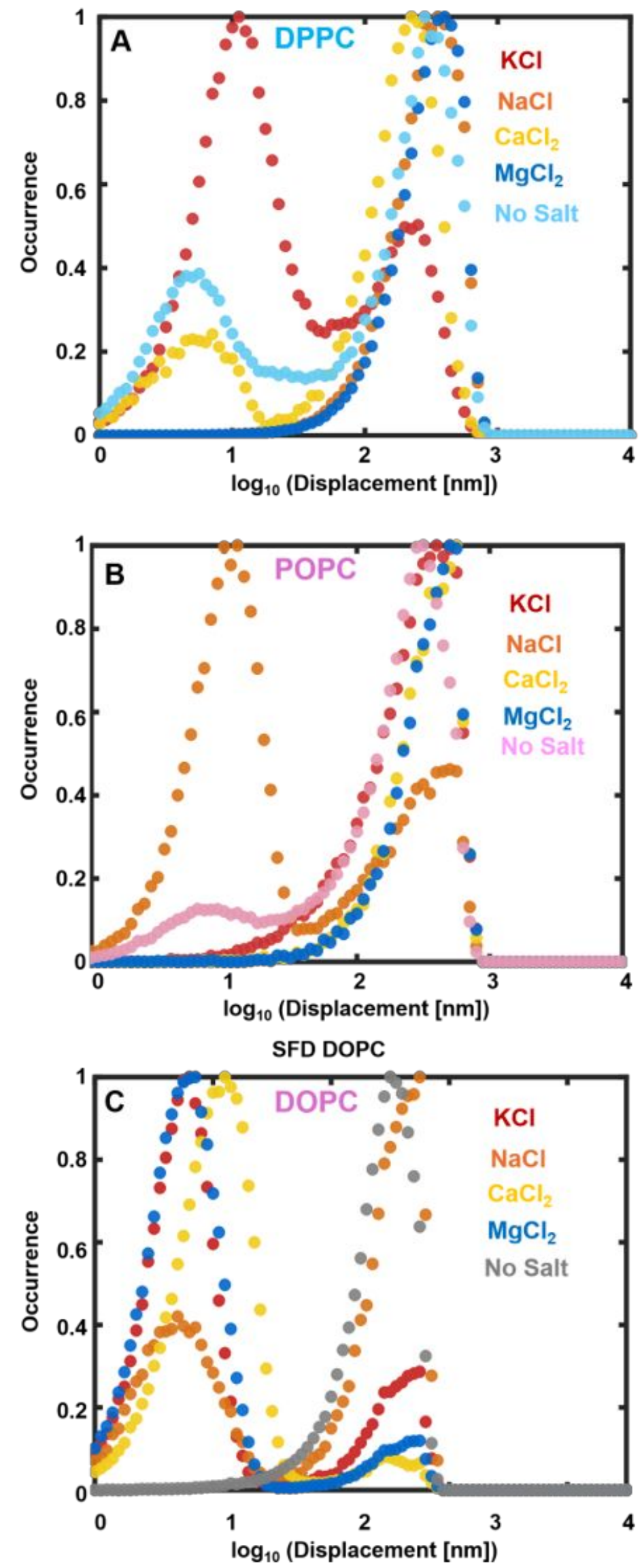

Figure S5: Single frame distributions for all salts in A) DPPC, B) POPC, and C) DOPC. No specific trend for the short or long displacement populations are observed in any conditions.

5. Schematic illustration of nanoparticle motion in salt free condition

The schematic illustrates the qualitative differences in nanoparticle dynamics across lipid systems and is intended as a conceptual aid rather than a quantitative representation of the data.

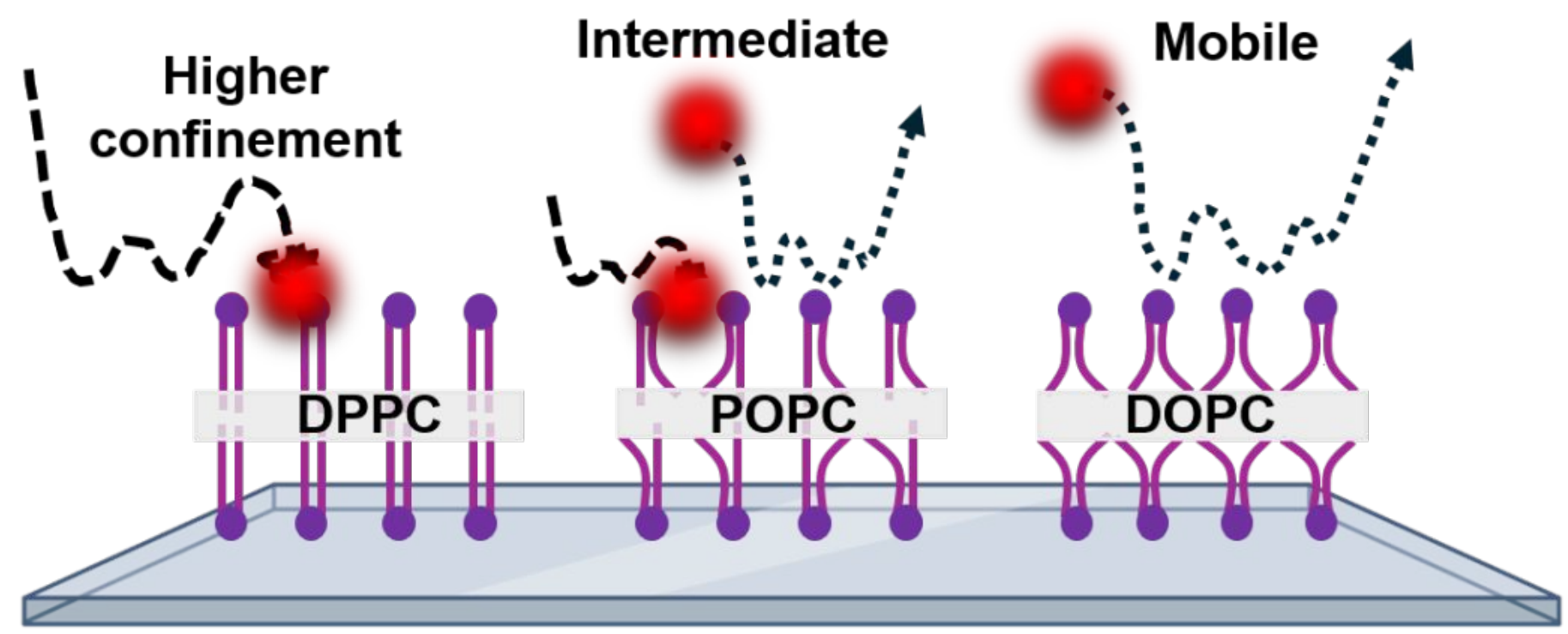

Figure S6. Schematic illustration of PS nanoparticle dynamics on supported lipid bilayers in salt-free conditions. Nanoparticles on gel-phase DPPC exhibit higher confinement, consistent with the distinct confined population observed in the single-frame displacement distribution (Figure 3A). Nanoparticles on POPC show intermediate trajectory behavior, while those on fluid-phase DOPC exhibit the greatest mobility, consistent with the broader van Hove displacement distribution observed for DOPC (Figure 3B). The schematic is a conceptual illustration and does not quantitatively represent trajectory dimensions or nanoparticle size.

6. Radius of Gyration for all salts

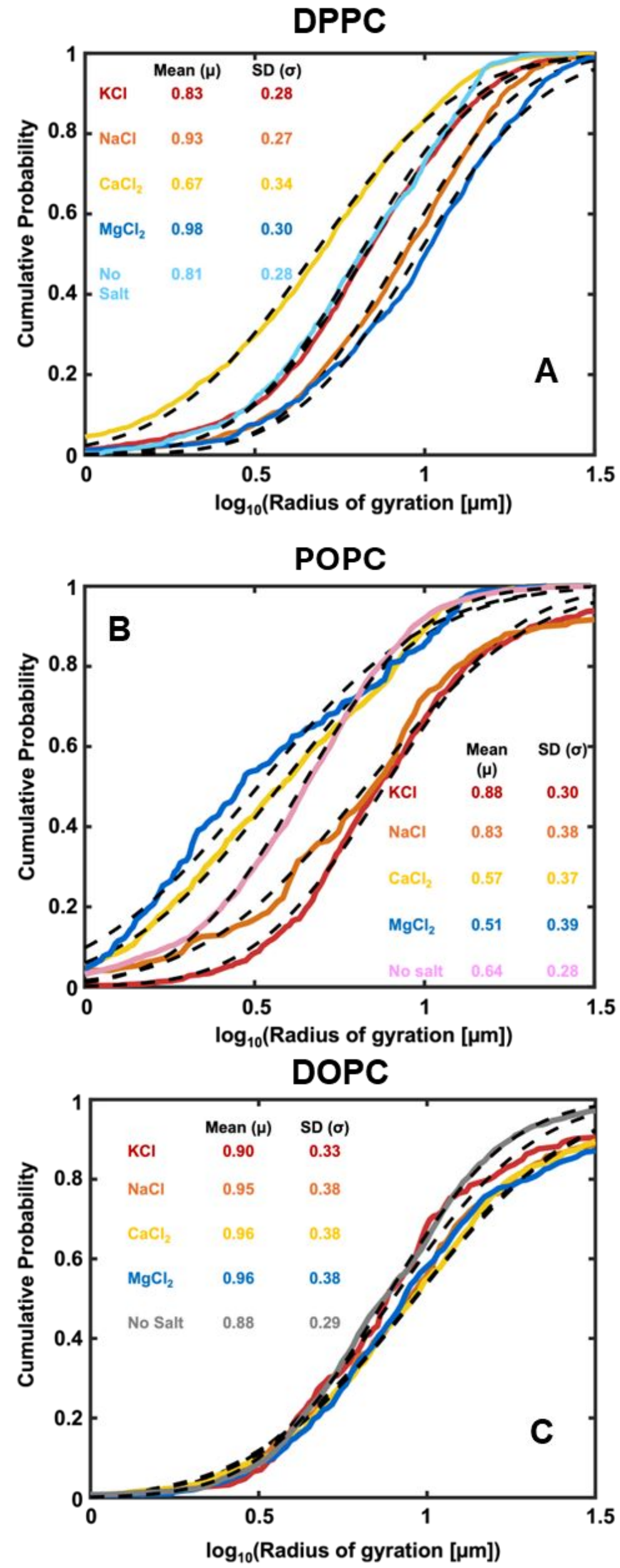

Figure S7. Salt-dependent RoG distributions for PS nanoparticles on DPPC (A), POPC (B), and DOPC (C) supported lipid bilayers. Cumulative distributions of $\log_{10}$(RoG/μm) under KCl, NaCl, $CaCl_2$, $MgCl_2$, and no-salt conditions. Mean and standard deviation values are indicated. Dashed lines are fits.

7. Representative fcsSOFI diffusion maps

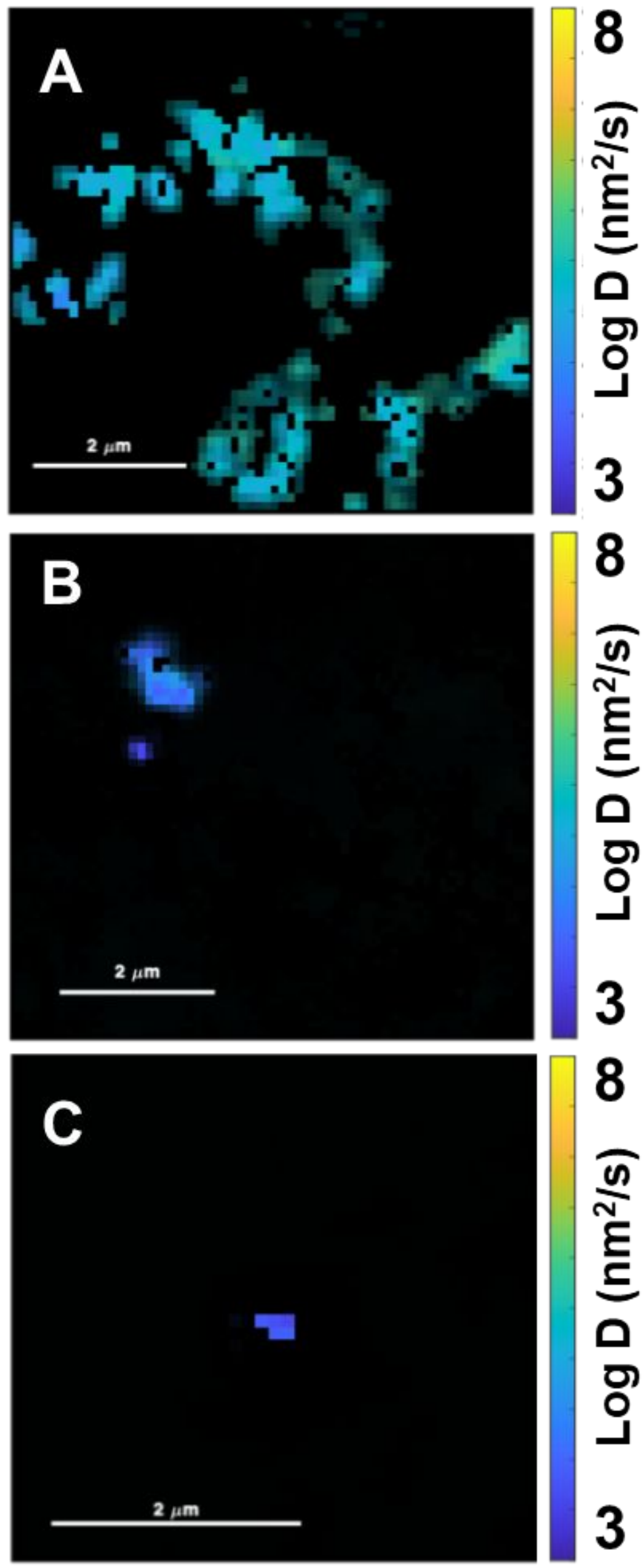

Figure S8. Representative fcsSOFI diffusion maps illustrating the three types of nanoparticle motion observed on supported lipid bilayers. A) Mobile nanoparticles exhibiting broad spatial distribution. B) Confined nanoparticles localized within a small region. C) Immobile nanoparticles. Color scale represents $\log_{10}$ (D/nm² s⁻¹).

8. Distribution of diffusion coefficients

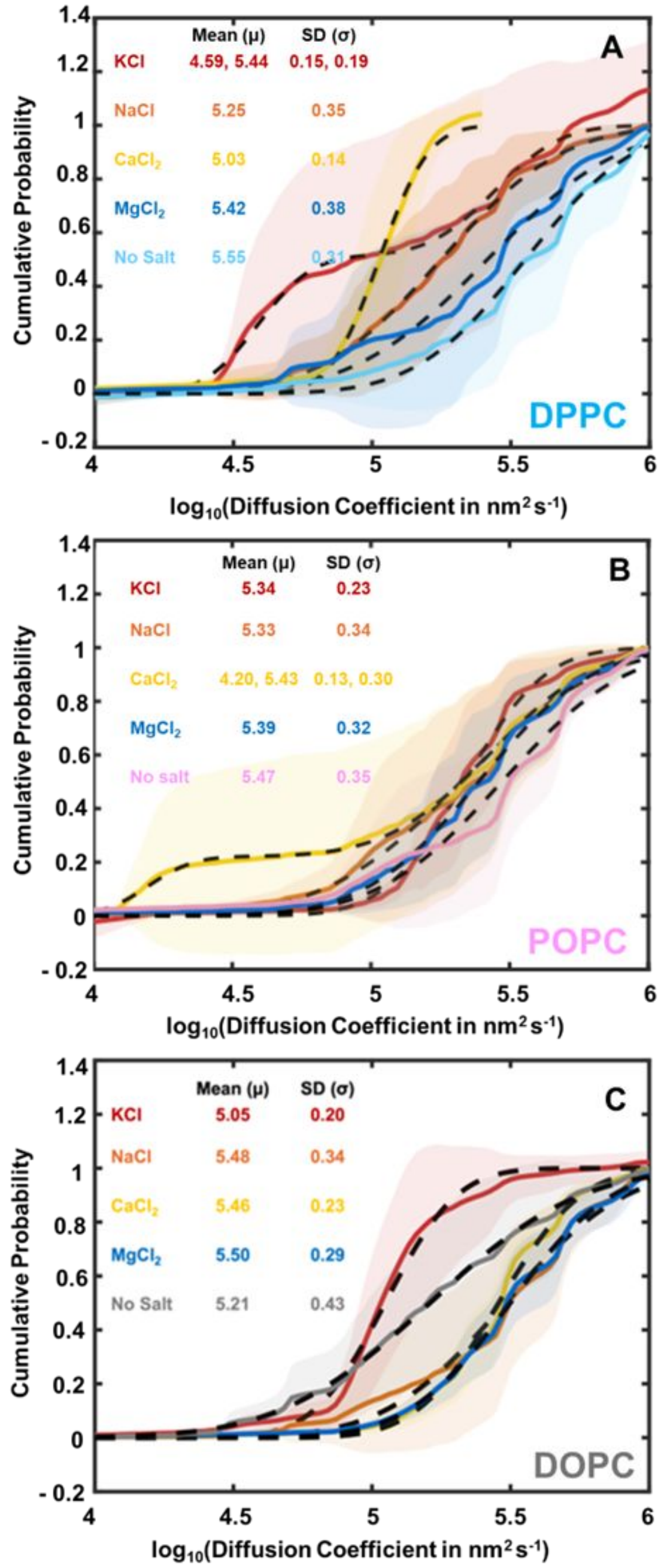

Figure S9. Salt-dependent diffusion coefficient distributions for PS nanoparticles on DPPC (A), POPC (B), and DOPC (C) supported lipid bilayers. Cumulative distributions of $\log_{10}(D/nm^2\ s^{-1})$ under KCl, NaCl, $CaCl_2$, $MgCl_2$, and no-salt conditions. Shaded regions indicate standard deviation. Mean and standard deviation values are indicated. Dashed lines are fits.

(1) Sil, D.; Osmanbasic, E.; Mandal, S. C.; Acharya, A.; Dutta, C. Variable Non-Gaussian Transport of Nanoplastic on Supported Lipid Bilayers in Saline Conditions. *J. Phys. Chem. Lett.* **2024**, *15* (20), 5428-5435.
